# Exosomes derived from myeloid-derived suppressor cells facilitate castration-resistant prostate cancer progression via S100A9/circMID1/miR-506-3p/MID1

**DOI:** 10.1186/s12967-022-03494-5

**Published:** 2022-08-02

**Authors:** Feng Gao, Qiaoping Xu, Zhe Tang, Nan Zhang, Yasheng Huang, Zhongyi Li, Yuliang Dai, Qiqi Yu, Jingyu Zhu

**Affiliations:** 1 Department of Urology, Hangzhou Hospital of Traditional Chinese Medicine, 453# Tiyuchang Road, Hangzhou, 310007 Zhejiang China; 2 Department of Clinical Pharmacology, Key Laboratory of Clinical Cancer Pharmacology and Toxicology Research of Zhejiang Province, Affiliated Hangzhou First People’s Hospital, Zhejiang University School of Medicine, Hangzhou, China; 3 Department of Urology, Second Affiliated Hospital, School of Medicine, Zhejiang University, 88# Jifanglu Road, Hangzhou, 310000 Zhejiang China; 4 Department of Clinical Laboratory, Zhejiang Provincial Hospital of Traditional Chinese Medicine, Hangzhou, China

**Keywords:** Castration-resistant prostate cancer (CRPC), Prostate cancer (PCa), Myeloid-Derived suppressor cells (MDSCs), Exosomes, Circular RNAs (circRNAs), miR-506-3p

## Abstract

**Background:**

Castration-resistant prostate cancer (CRPC) is a major cause of recurrence and mortality among prostate cancer (PCa) patients. Myeloid-derived suppressor cells (MDSCs) regulate castration resistance in PCa. Previously, it was shown that intercellular communication was efficiently mediated by exosomes (Exos), but the role and the mechanism of MDSC-derived Exos in CRPC progression was unclear.

**Methods:**

In this study, the circRNA expression profiles in PC3 cells treated with MDSC-Exo and control cells were investigated using a circRNA microarray.

**Results:**

The data showed that circMID1 (hsa_circ_0007718) expression was elevated in PC3 cells treated with MDSC-Exo. Moreover, high circMID1 expression was found in PCa compared with benign prostatic hyperplasia (BPH) tissues and in CRPC patients compared with hormone sensitive prostate cancer (HSPC) patients. Further studies showed that MDSC-Exo accelerated PCa cell proliferation, migration, and invasion, while circMID1 deficiency inhibited MDSC-Exo-regulated CRPC progression in vitro and in vivo*.* Mechanistically, MDSC-derived exosomal S100A9 increased circMID1 expression to sponge miR-506-3p, leading to increased MID1 expression and accelerated tumor progression.

**Conclusion:**

Together, our results showed that a S100A9/circMID1/miR-506-3p/MID1 axis existed in MDSC-Exo-regulated CRPC progression, which provided novel insights into MDSC-Exo regulatory mechanisms in CRPC progression.

**Supplementary Information:**

The online version contains supplementary material available at 10.1186/s12967-022-03494-5.

## Background

Prostate cancer (PCa) is a malignancy, which usually occurs among males in Western countries [1]. Although PCa was originally responsive to androgen deprivation therapy (ADT), the occurrence of castration-resistant prostate cancer (CRPC) following ADT is a major clinical problem because the recurrent disease does not respond well to alternative therapies [2]. The prognoses of CRPC patients remain poor, and CRPC is not considered curative [3–5]. Consequently, identifying new molecular mechanisms of androgen-independent signalling pathways could identify more effective therapies for CRPC.

Cancer cells might weaken the immune defense by activating immunosuppressive mechanisms or inhibiting immune responses, which form a tumor microenvironment leading to cancer cell growth, survival, and metastatic spread. Myeloid-derived suppressor cells (MDSCs) comprise a prominent immune cell subset infiltrating the CRPC microenvironment [6, 7]. Elevated levels of MDSCs are detected in tumor tissue and peripheral blood of PCa patients, and the levels correlate with disease progression [8, 9]. MDSCs are immature myeloid cells with potent immunosuppressive mechanisms in tumor microenvironments (TME). They mediate immune suppression by polarizing macrophages to a tumor-promoting phenotype [10] and preventing tumor-reactive T lymphocyte activation, which promote tumor angiogenesis [11]. Among these mechanisms, some demand interactions between target cells and MDSCs, or soluble mediator release. IL23 produced by MDSCs can activate the androgen receptor (AR) pathway in PCa cells, which promote cell proliferation and survival in androgen-deprived conditions. Antibody-mediated IL-23 inactivation restores sensitivity to ADT in mice, suggesting a MDSC-mediated castration resistance mechanism [6]. However, the MDSCs-mediated resistance mechanism to castration remains largely unknown.

It is accepted that intercellular communication is not related to soluble mediators or cell-to-cell contact, and could instead be effectively mediated by exosomes (Exos) [12, 13]. Exos derived from immune cells are major components of the TME, which lead to heterogeneous and complex interplay between tumor cells and immune cells. In fact, MDSCs-shed Exos contain proinflammatory mediators such as S100A9 and S100A8 [14], which promote tumor progression and suppress anti-tumor responses. Recent reports have shown that Exos derived from MDSCs had the biological ability to regulate chemotherapy resistance and tumor progression [15]. Despite an increasing number of investigations indicating MDSC-released exosomes function in immune suppression, it is not clear if MDSCs-derived Exos have specific biological activities regarding castration resistance and the regulation of prostate tumor progression. Identifying new molecular mechanisms of MDSC-Exo-regulated CRPC progression may therefore provide promising ways to improve CRPC treatment. In addition, exosomes are emerging as a new type of cancer biomarkers. Numerous studies have now shown that the unequivocal markers for tumor-derived exosomes may represent a very sensitive diagnostic approach for clinical evaluation of disease progression [16–18]. Rashid's research has shown that the differential in vivo distribution of radioisotope ^131^I-labeled exosomes from diverse cellular origins, e.g., tumor cells with or without treatments, MDSCs and endothelial progenitor cells [19]. Alterations in protein or nucleic acid profiles of exosomes are correlate with pathological processes of many diseases including cancer, thus, tracking this change could be utilized to monitor disease progression, metastasis, and exosome-based targeted therapy.

Circular RNAs (circRNAs) have been recently shown to be endogenous noncoding RNAs, which function significantly in many cancer progressions, including Pca [20, 21]. One of the main ways that circRNAs regulate gene expression is by functioning as a miRNA “sponge” to modify miRNA activity via sequestration, which alters mRNA target gene expression [22]. The circRNAs from the *AR* gene have been identified in CRPC [23], which suggests a potential role as biomarkers or as therapeutic targets. Wu et al. reported that circRNA17 altered the enzalutamide sensitivity and CRPC cell invasion as suppressors, by regulating Arv7 expression by sponging miR-181c-5p [24]. Nevertheless, the functions and roles of circRNAs in PCa progression are still unknown.

In the current study, using a circRNA microarray profiling, we showed that circMID1 (hsa_circ_0007718) expression was markedly elevated in PC3 cells treated with Exos from MDSCs. Moreover, high circMID1 expression was found in PCa compared with benign prostatic hyperplasia tissues and in CRPC patients compared with hormone sensitive prostate cancer (HSPC) patients*. *In vitro and in vivo studies showed that circMID1 deficiency inhibited MDSC-Exo-regulated CRPC progression. Mechanistically, we showed that MDSC-derived exosomal S100A9 increased circMID1 expression to sponge miR-506-3p, which might then lead to increased MID1 expression and accelerate tumor progression. The results provided novel insights into the MDSC-Exo regulatory mechanisms in CRPC progression.

## Methods

### Cell culture

An androgen-independent human PCa cell line (PC3) and DU145 were obtained from the American Type Culture Collection (ATCC, Manassas, VA, USA) and maintained according to ATCC guidelines. We cultured PC3 cells in RPMI-1640 medium with 100 IU/mL penicillin, 10% fetal bovine serum (FBS), and 100 μg/mL of streptomycin, and maintained the cells in a humidified incubator with an atmosphere comprised of 5% CO_2_ and 95% air at 37 °C.

### Isolation and characterization of Exos derived from MDSCs

We generated human MDSCs from human peripheral blood mononuclear cells (PBMCs) in vitro as previously described [6, 25]. Briefly, we isolated PBMCs from healthy donors by venipuncture, followed by differential density gradient separation (Ficoll HyPaque; Sigma-Aldrich, St. Louis, MO, USA). The PBMCs (1 × 10^6^ cells/mL) were cultured in T25 flasks with RPMI 1640 medium containing 10% heat-inactivated FBS with granulocyte–macrophage colony-stimulating factor (PeproTech, Rocky Hill, NJ, USA) at 10 ng/mL and IL-6 at 10 ng/mL for 1 week. In vitro-generated polymorphonuclear MDSCs (PMN-MDSCs) were characterized by flow cytometry for expressions of CD15, CD11b, and CD14. The PMN-MDSCs were CD15^+^ CD14^−/low^ CD11b^+^ (the sorting strategy is indicated by green boxes in Additional file 1: Fig S1A). We collected and used the cells for further experiments.

We cultured isolated MDSCs (2 × 10^6^ cells/mL) in RPMI-1640 medium containing 10% exosome-depleted FBS for 2 days. FBS was depleted from contaminating bovine exosomes by ultracentrifugation for at least half of a day at 100,000×*g*. We collected the culture medium using a 0.22 μm filter (BD Falcon; Corning, Corning, NY, USA), and extracted Exos via the ExoQuick Exosome Precipitation Solution (System Biosciences, Palo Alto, CA, USA) following standard procedures. Exos were resuspended in 200 μL of medium. Electron micrographs were observed by transmission electron microscopy (Tecnai-12; Philips, Amsterdam, Netherlands). The BCA protein assay kit was used to quantify the exosomes. Protein markers, CD9, CD63, Hap70, calnexin were determined by immunoblotting. The size and concentration of exosomes were directly measured using the NanoparticleTracking Analyzer (NTA, Malvern Panalytical, Malvern, UK). Identification of MDSC-exosomes was shown in Additional file 1: Fig. S1.

### Microarray analysis of circRNAs

We performed circRNA microarray analysis using the Arraystar Human circRNA Array, version 2.0 (Arraystar, Rockville, MD, USA). We performed RNA extraction and microarray hybridization following standard procedures. We extracted total RNA from PC3 cells treated with phosphate-buffered saline (PBS) or MDSC-derived Exos (50 μg/mL) at 48-h using TRIzol reagent (Life Technologies, Carlsbad, CA, USA) following standard procedures. We digested total RNA using RNAse R (Epicentre, Madison, WI, USA) to eliminate linear RNAs and enrich circRNAs. Then, the enriched circRNAs was amplified and transcribed into fluorescent cRNAs using the random priming method using the Arraystar Super RNA Labeling Kit (Arraystar), and hybridizing the cRNAs using the Arraystar Human circRNA Array, version 2 (8 × 15 K). Finally, the slides were washed and scanned using a Scanner G2505C (Agilent, Santa Clara, CA, USA) and the acquired array images were analyzed. Quantile normalization and subsequent data processing was performed using the R software limma package. CircRNAs differentially expressed with statistical significance between MDSC-Exo-PC3 and control-PBS-PC3 (fold-change (FC) ≥ 2 and p ≤ 0.05) were identified through Volcano Plot filtering. Hierarchical clustering was performed to show the distinguishable expression pattern of circRNAs among samples.

### Clinical prostate specimens

Patients (ages ranged from 49 to 78 years with a median age of 64 years) undergoing radical prostatectomy for localized PCa (n = 56) and benign prostatic hyperplasia (n = 31) undergoing transurethral resection of the prostate were collected from the Hangzhou Hospital of Traditional Chinese Medicine. All tissue specimens were pathologically confirmed as PCa by two experienced pathologists. The Ethics Committee of the Hangzhou Hospital of Traditional Chinese Medicine approved this study, and we obtained written consent from each participant prior to the study.

### Quantitative real-time PCR

We extracted RNA and miRNAs from the cultured cells and tissues using TRIzol reagent (Invitrogen, Karlsruhe, Germany) and an mirVana miRNA isolation kit (Ambion, Austin, TX, USA) following standard instructions. We then synthesized cDNAs using a High Capacity cDNA Reverse Transcription Kit (Thermo Fisher Scientific, Waltham, MA, USA). We performed qPCR analyses using an ABI PRISM 7500 Sequence Detection System (Life Technologies, Grand Island, NY, USA). We used U6 snRNA as an endogenous control of miRNAs, and normalized circRNAs levels to glyceraldehyde 3-phosphate dehydrogenase (GAPDH). We calculated relative gene expression levels using the 2^−ΔΔCt^ method. The primers used for RT-PCR are included in Table 1.

**Table 1:** Primers used in real-time RT-PCR analysis

Primer	Sequence (5′ to 3′)
has_circ_0009154-F	ATTAGTTCCGTCACAGGGGC
has_circ_0009154-R	AGGTCCTGCTATTTCTCCTCT
has_circ_0085361-F	GGATGAATCCCAGTCCCTGT
has_circ_0085361-R	GCCTTCACTTGCAACGTTTC
has_circ_0007718-F	TGGCTAAACTCATCCAAACCTG
has_circ_0007718-R	GCAAAACCCAAGGAAGCTGA
has_circ_0001944-F	GGGAAGACTTGGTTGTGCAG
has_circ_0001944-R	GTGTTTGCAAGCCAGGTACA
has_circ_0026782-F	TCCCCTGATAGCCACTACCT
has_circ_0026782-R	CTTGCCTCATATCGGTGTGC
has_circ_0018064-F	GGCGGTCCCTCATCAAGAA
has_circ_0018064-R	CTCAGCTTGGCTCGTTCATC
has_circ_0086414-F	ATGCTGCATTCCCCTCTCG
has_circ_0086414-R	TTCTCGAGACATGATGGCCC
has_circ_0004119-F	TGAGGATCCAGAACTAACGCA
has_circ_0004119-R	TTCCAACTGCTCCATTCCCT
MID1-F	GCTGGGGGAGAAAAGGTTGA
MID1-R	AAAAGCAACCCGGCGAAATC
GAPDH-F	TCGGAGTCAACGGATTTGGT
GAPDH-R	TTCCCGTTCTCAGCCTTGAC
U6-F	CTCGCTTCGGCAGCACA
U6-R	AACGCTTCACGAATTTGCGT
miR-506-3p-F	ACACTCATAAGGCACCCTTC
miR-506-3p-R	TCTACTCAGAAGGGGAGTAC

### Cell transfection

Small interfering RNAs (siRNAs) targeting human circMID1 (si-circMID1), MID1 (si-MID1), and nonspecific negative control oligos (si-control), miR-506-3p-mimics, and the corresponding negative control mimic (miR-NC), anti-miR-506-3p and anti-miR-ctl plasmid-mediated overexpressing circMID1 (over-circMID1) or MID1 (over-MID1) vector, and siRNA targeting S100A9 (si-S100A9) were obtained from Gene Pharma (Shanghai, China). The lentivirus targeting circMID1 (sh-circMID1) was purchased from GeneChem (Shanghai, China). We performed transfections using Lipofectamine 3000 (Invitrogen) following the standard instructions. Overexpression or knockdown effects were measured by RT-qPCR using RNA extracted 2 days after transfection.

For treatments, we preincubated PC3 cells with si-control, si-circMID1, or si-MID1, and anti-miR-ctl, or anti-miR- 506-3p, and miR-NC, or miR-506-3p, over-circMID1 or over-MID1 for 30 min and then treated them with or without MDSC-derived Exos (50 μg/mL) for the indicated times.

### Cell proliferation assay

We assessed cell proliferation using the Cell Counting Kit-8 (CCK-8) assay (Dojindo, Shanghai, China). In brief, we seeded cells (3 × 10^4^) into plates with 96 wells and incubated them at 37 °C for 1 day before transfection. We added CCK-8 solution (10 μL) to every well 2 days after transfection. At the indicated time points, we measured absorbance at a wavelength of 450 nm for 5 days using a Spectra Max 250 spectrophotometer (Molecular Devices, San Jose, CA, USA). The experiments were performed in triplicate.

### Migration and invasion assays

We performed Transwell assays for PC3 and DU145 cell invasion and migration in 8.0 µm Boyden chambers with 24 wells. For the invasion assays, the chambers were precoated with Matrigel, and cells (3 × 10^4^) were seeded in serum-free medium into the upper level of the chamber. We added full culture medium to the lower chamber as a chemoattractant. After 1 day of incubating at 37 °C with 5% CO_2_, the membranes were fixed with methanol and stained with 0.1% Crystal Violet. We randomly selected five visual fields (200×) and counted the number of the invading cells using a light microscope. We also carried out migration assays without precoating the chambers.

### Cellular uptake of exosomes

Exosomes (20 mg) derived from MDSCs were labeled with CFSE dye, which was diluted at a ratio of 1:1000. After 15 min incubation at 37 °C, the exosomes were harvested and washed with PBS by centrifugation (100,000×*g*, 70 min). CFSE-labeled exosomes or unlabeled exosomes were added to PC3 and DU145 cells and incubated for 48 h at 37 °C, and the cellular uptake was observed using a confocal fluorescence microscope. The sample was treated in the same manner and used as a control for any free CFSE dye left in the solution. Immunofluorescence microscopy was done as previously described [26]. Primary antibody was mouse IgG anti-alpha-tubulin DM1A (1:2000) (Sigma). Secondary antibody was donkey anti-mouse IgG AlexaFluor 594 (1:500) (Molecular Probes).

### Luciferase reporter assay

The constructs containing wild-type (WT) or mutant (MUT) cirMID1 sequences or the MID1 3'-UTR containing the WT or mutated miR-506-3p binding site were subcloned into the luciferase gene by using a pmir-GLO vector (Promega, Madison, WI, USA). We co-transfected HEK293 or PCa cells with 100 ng of luciferase reporter vectors combined with miRNAs mimics/NC or a miR-506-3p mimics/NC (200 pmol) using Lipofectamine 2000 (Invitrogen and Thermo Fisher Scientific). After 2 days of transfection, we detected firefly and Renilla luciferase activities using a dual luciferase reporter assay system (Promega), and expressed the specific activity as the relative activity of firefly to Renilla luciferase. The experiments were conducted in triplicate.

### RNA immunoprecipitation (RIP) assays

We performed RIP assays using a Magna RIP RNA-Binding Protein Immunoprecipitation Kit (Millipore, Bedford, MA, USA) following the standard protocol. PC3 cells (2 × 10^7^) were lysed in RIP lysis buffer and the cell lysates incubated, dividing them into two equivalent parts, with either isotype-matched anti-IgG antibody (Millipore) or 5 μg human anti-Argonaute2 (AGO2) antibody (Millipore) with rotation at 4 °C overnight. We added magnetic beads and continued incubation at 4 °C for 1 h. We immunoprecipitated the samples with proteinase K at 55 °C for 1 h, then treated the complexes of RNA with TRIzol reagent (Life Technologies) for further purification. We utilized purified RNA to detect circMID1 and miR-506-3p expression levels using qRT-PCR.

### Western blot analysis

For western blots, in brief, we collected cells from various treatment groups and lysed them in RIPA buffer (Beyotime Biotechnology, Nantong, China) including 1% protease inhibitor (Cell Signaling Technology). The protein concentration was measured using the BCA Protein Assay kit (Beyotime Biotechnology), and total protein (50 μg) was separated using sodium dodecyl sulphate polyacrylamide gel electrophoresis (SDS-PAGE; 10%) and transferred to polyvinylidene difluoride (PVDF) membranes (Millipore). After blocking with 5% skim milk in Tris-buffered saline-Tween (TBST) buffer under room temperature for 1 h, we incubated the membranes with primary antibody against S100A9, DHX9, QKI, MID1 (1:1000; Abcam, Cambridge, MA, USA), for 1 day at 4 °C, followed by incubation with HRP-conjugated secondary antibody (1:5000; Rockland, Limerick, PA, USA) at room temperature for 1 h. Anti-GAPDH antibody (1:1000; Affinity, Cambridge, MA, USA), β-actin or Hsp70 (1:1000; Abcam) measured GAPDH, β-actin or Hsp70 as protein loading controls. We visualized the blots using a chemiluminescent reaction system (ECL; Beyotime, Shanghai, China).

### Mouse xenograft model assays

All male BALB/c (nu/nu) mice were purchased from the Laboratory Animal Center of Zhejiang University. Animals were maintained in standard conditions with water and food provided ad libitum. The Institutional Animal Care and Use Committee at of the Second Affiliated Hospital in Zhejiang University approved the animal studies. For xenograft animal assays, 4 ~ 6-week-old BALB/c nude mice were injected subcutaneously with a total of 5 × 10^6^ cells into the right flank (five mice per group). We measured the tumor size every 5 days for 1 month, and the volume was determined using the formula: volume = (length × width^2^) × 0.5. After 1 month, we euthanized the mice by carbon dioxide asphyxiation and removed the tumors and weighed them. Finally, the tumor tissues were immediately flash-frozen in liquid nitrogen and stored at − 80 °C until analysis.

### Immunohistochemistry (IHC) staining

The tumors were resected and fixed in 4% paraformaldehyde, embedded with paraffin, and cut into 5 μm sections. The slices were dewaxed with xylene and dehydrated in graded ethanol, followed by blocking endogenous peroxidase with 3% hydrogen peroxide. After antigen retrieval, the sections were incubated with primary antibodies against MMP-2 (1:1000; Abcam, Cambridge, UK) or Ki67 (1:1000; Abcam) overnight at 4 °C. Then, the slides were incubated with horseradish peroxidase-labeled secondary antibody (1:1000, Abcam) for 1 h. The slides were developed with a diaminobenzidine (DAB) detection kit (Dako Cytomation, Glustrop, Denmark). After being counterstained with hematoxylin, the samples were observed using a light microscope (Olympus, Tokyo, Japan).

### Statistical analysis

Data are denoted as the mean ± standard deviation (SD). Student’s *t*-test was used to compare two groups. Comparisons of data among multiple groups were analyzed using one-way analysis of variance (ANOVA), followed by Bonferroni post hoc test. We calculated correlations through Pearson's correlation. P-values < 0.05 indicated a statistical significance. We performed all experiments at least in triplicate.

## Results

### Expression profiles of circRNAs in PC3 cells treated with exosomes derived from myeloid-derived suppressor cells

**Fig. 1:**
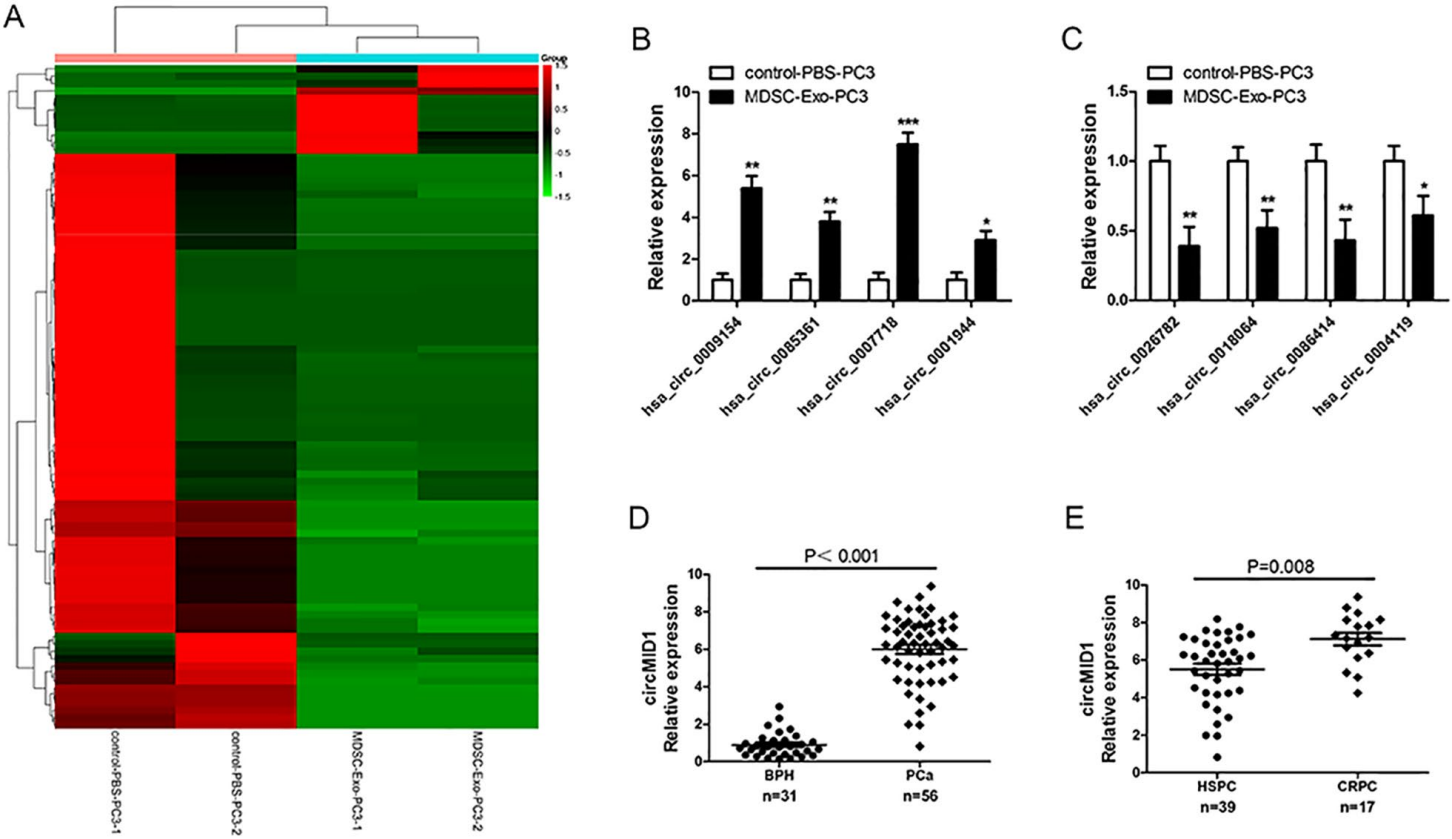
The circRNA expression profiles in MDSC-Exo-PC3 cells. **A** Hierarchical clustering analysis of circRNAs that were differentially expressed between MDSC-Exo-PC3 cells and control-PBS-PC3 cells. The red color represents high expression levels, whereas the green color represents low expression levels. **B**, **C** The relative expression of the four most upregulated (**B**) and four most downregulated circRNAs (**C**) were validated by qRT-PCR in MDSC-Exo-PC3 cells and control-PBS-PC3 cells. ^*^P < 0.05; ^**^P < 0.01; ^***^P < 0.001 vs. control-PBS-PC3. **D** Relative expression of circMID1 in benign prostatic hyperplasia (BPH; n = 31) and PCa tissues (n = 56) determined by qRT-PCR. **E** The circMID1 expression was detected between HSPC (n = 39) and castration-resistant prostate cancer (CRPC; n = 17) by qRT-PCR. All the experiments were performed at least three times. MDSC: myeloid derived suppressor cells

To determine the role and molecular mechanisms underlying MDSC-Exo in PCa progression, PC3 cells were co-cultured with MDSC-Exo or control-PBS and then PC3 cells were collected for their circRNA profiles analysis by using circRNA microarrays. We detected a total of 4248 circRNAs. Among them, 90 circRNAs were differentially expressed (fold-changes > 2.0; P < 0.05 and FDR < 0.05) between MDSC-Exo-PC3 cells and control-PBS-PC3 cells (Fig. 1A). Of the 90 circRNAs, 12 circRNAs were upregulated and 78 were downregulated in MDSC-Exo-PC3 cells compared with controls. To further evaluate the microarray data, we selected the four most upregulated and four most downregulated circRNAs to confirm their expressions in PC3 cells with different treatments by using qRT-PCR (Fig. 1B, C). The hsa_circ_0007718 was the top upregulated circRNA in MDSC-Exo-PC3 cells. By browsing the hg19/GRCh37 reference genome, hsa_circ_0007718, located at chrX:10491131–10535643, was assumed to derive from the *MID1* gene (midline 1), which is located on chromosome Xp22.2. In this manner, we termed hsa_circ_0007718 as “circMID1.”

To confirm that circMID1 was differentially expressed in PCa, we examined prostatectomy specimens of PCa patients and benign prostatic hyperplasia (BPH) using qRT-PCR. We discovered that the circMID1 expression was significantly higher in PCa compared to BPH tissues (p < 0.01, Fig. 1D). The circMID1 expression was also higher in CRPC patients compared to HSPC patients (p = 0.008, Fig. 1E). In summary, the results suggested that circMID1 upregulation might be relevant to PCa progression.

### CircMID1 deficiency inhibited myeloid-derived suppressor cells (MDSC)-exosome (Exo)-treated PC3 cell proliferation, invasion, and migration

**Fig. 2:**
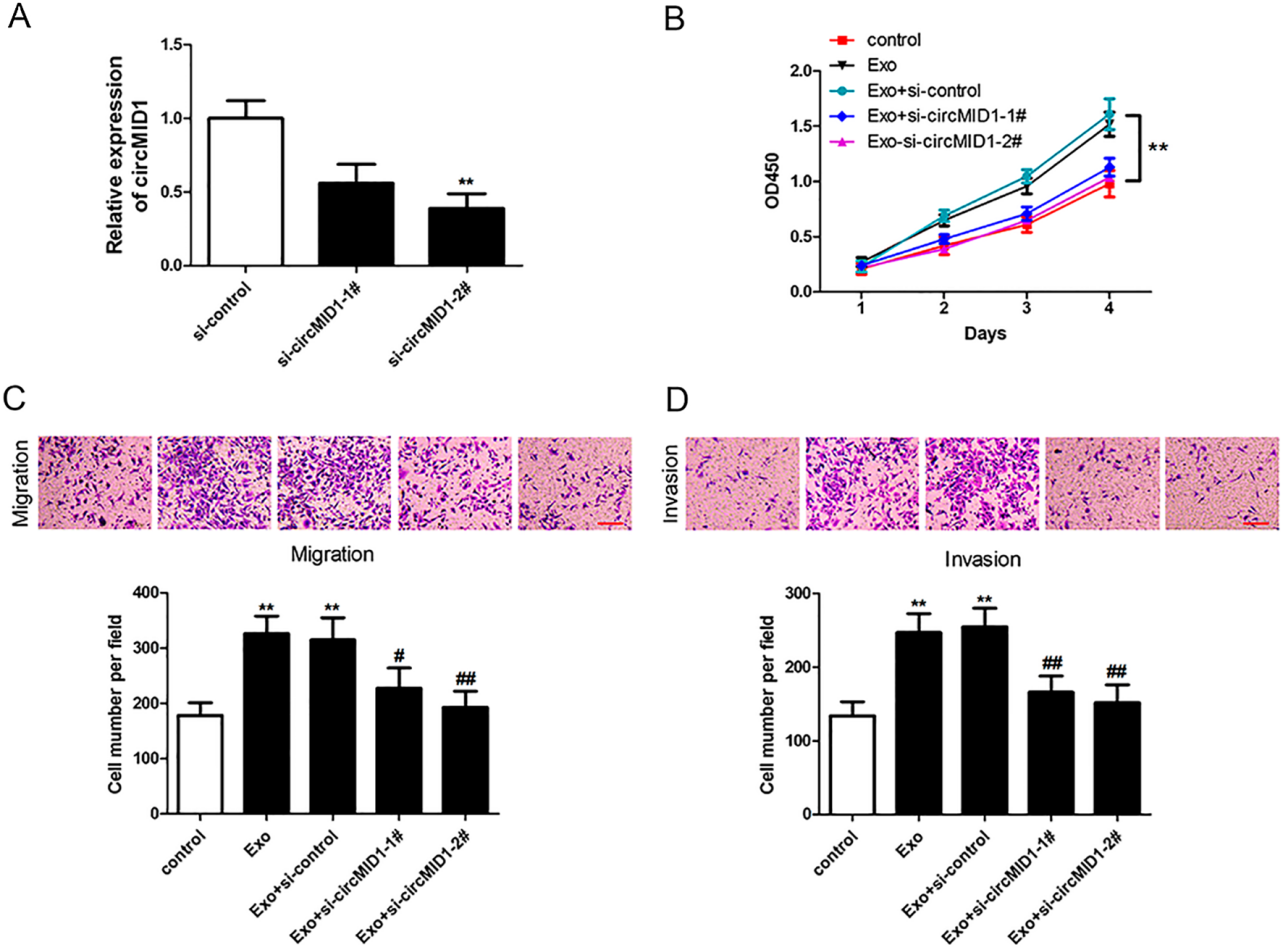
CircMID1 knockdown inhibits cell proliferation, invasion, and migration in MDSC-Exo-treated PC3 cells. **A** Relative expression of circMID1 in PC3 cells transfected with specific siRNAs against circMID1 (^**^p < 0.01). **B** CCK-8 assays were used to measure cell proliferation in control PC3 cells and MDSC-Exo-PC3 cells transfected with si-control or si-circMID1 (^**^P < 0.01). **C**, **D** Transwell assays were used to determine cell migration (**C**) and invasion (**D**) in control PC3 cells and MDSC-Exo-PC3 cells transfected with si-control or si-circMID1. Scale bar, 100 μM. ^*^, ^#^P < 0.05; ^**^, ^##^P < 0.01, ^*^ vs. control, ^#^ vs. Exo. All the experiments were repeated at least three times

Because circMID1 was upregulated in MDSC-Exo-PC3 cells, we utilized RNA interference to knockdown circMID1 expression and validate its biological mechanisms during MDSC-Exo-regulated CRPC propagation. The qRT-PCR analyses showed that circMID1 expression in the PC3 cell lines was successfully inhibited by siRNAs (Fig. 2A). In vitro investigations showed that MDSC-Exo promoted the PC3 cell migration, proliferation, and invasion (Fig. 2B–D). However, CCK-8 assays detected that circMID1 downregulation significantly suppressed PC3 cell proliferation induced by MDSC-Exo (Fig. 2B). Next, we assessed the circMID1 role in prostate cancer migration and invasion using Transwell assays. The circMID1 knockdown inhibited PC3 cell invasion and migration induced by MDSC-Exo (Fig. 2C, D).

### Exosomal S100A9 from MDSC promoted circMID1 expression in PC3 cells

**Fig. 3:**
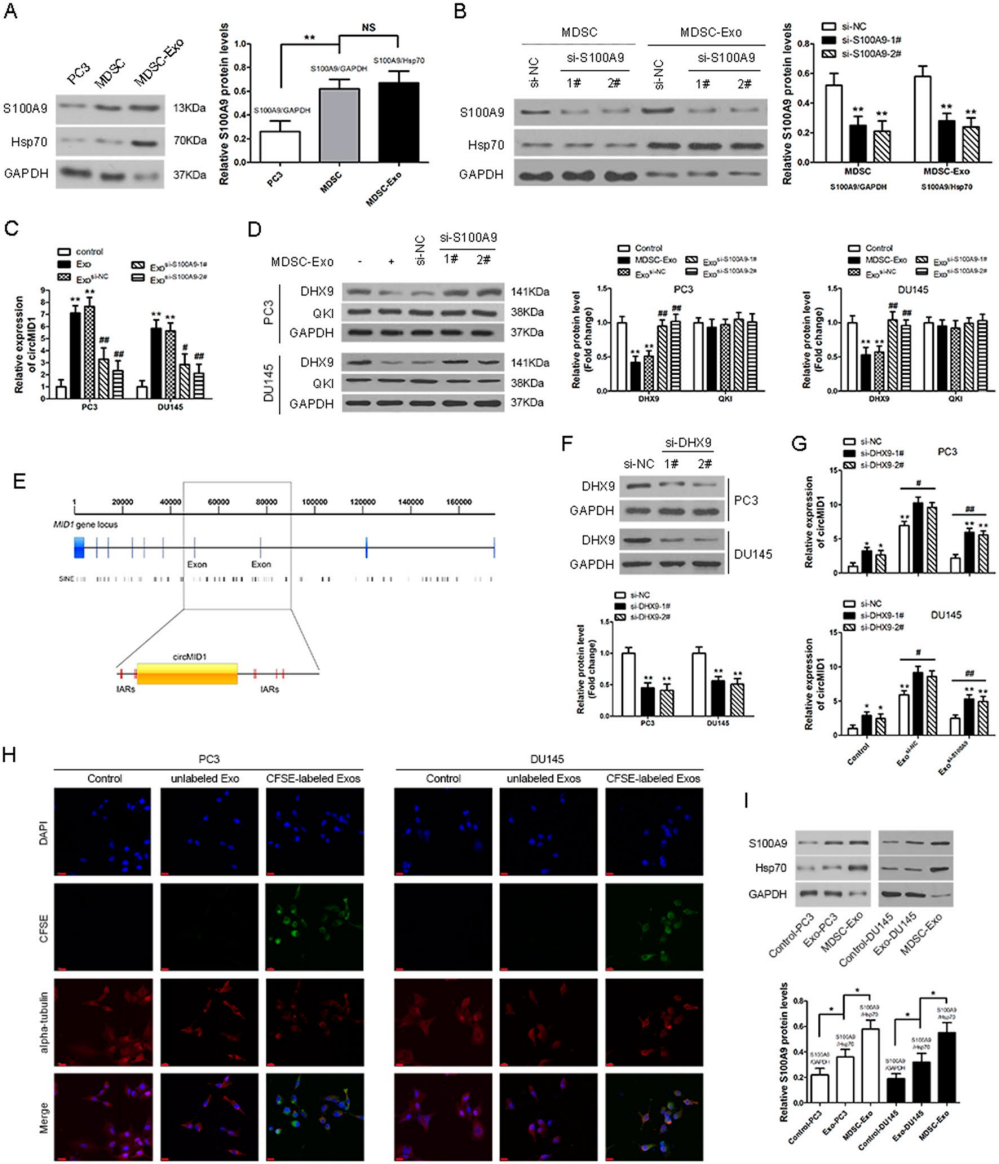
Exosomal S100A9 from MDSC promoted circMID1 expression in PC3 cells. **A** A representative western blot of S100A9 expression in MDSC-Exo, and quantitative analysis of S100A9 expression. GAPDH is control protein for cells, Hsp70 is control protein for exosomes. **P < 0.01, NS: no significance. **B** A representative western blot of S100A9 expression in MDSCs and MDSC-Exo treated with control siRNA (si-NC) or S100A9 siRNA (si-S100A9), and quantitative analysis of S100A9 expression. **P < 0.01, ^*^ vs. si-NC. **C** Relative expression of circMID1 in PC3 and DU145 cells treated with MDSC-Exo or MDSC-Exo^si-S100A9^. ^**^, ^##^ P < 0.01, ^*^vs. control, ^#^ vs. Exo. **D** The expressions of RNA-binding protein DHX9 and Quaking (QKI) were determined by western blotting in PC3 and DU145 cells treated with MDSC-Exo or MDSC-Exo^si-S100A9^. The analysis of result of western blot for DHX9 and QKI. ^**^, ^##^P < 0.01, ^*^vs. control, ^#^ vs. Exo. **E** The distribution of IARs within the *MID1* gene locus. The upper line shows the whole length of MID1, while the lower line shows ± 500 bp of the region between exon 7 and exon 8. **F** The expression levels of DHX9 were evaluated by western blotting after transfection with DHX9 siRNAs in PC3 and DU145 cells, and quantitative analysis of S100A9 expression. **P < 0.01, ^*^ vs. si-NC. **G** The relative levels of circMID1 in MDSC-Exo-treated PC3 and DU145 cells were measured after DHX9 was silenced. ^*^, ^#^P < 0.05; ^**^, ^##^P < 0.01, ^*^ vs. control + si-NC. **H** The uptake of MDSC-derived exosomes by PC3 and DU145 cells at 48 h. The free CFSE treated cells was used as a control (Control). Green, CFSE-labeled exosomes; red, alpha-tubulin, a microtubule marker (scale bar, 20 μm). **I** The level of S100A9 in PC3 and DU145 cells after 48-h incubation with MDSC-Exo was determined by western blotting. The analysis of result of western blot for S100A9. ^*^P < 0.05

We next investigated the underlying mechanism of the circMID1 increase in PC3 cells. The proinflammatory protein, S100A9, was previously shown to be abundant in MDSC-shed exosomes and to have a chemotactic function for MDSCs [27]. As shown in Fig. 3A, abundant S100A9 protein was detected in MDSC-Exo. Then, S100A9 expression was knocked down in MDSCs using siRNA. MDSC-Exo^si-S100A9^, which had a lower expression of S100A9 in exosomes, was isolated and purified (Fig. 3B). The knockdown of S100A9 significantly reduced circMID1 expression levels in MDSC-Exo-treated PC3 and DU145 cells (Fig. 3C). As RNA binding proteins (RBPs) are necessary for circRNA [28], we then examined whether RBPs DHX9 and QKI, which regulate the biogenesis of a number of circRNAs [29, 30], were responsible for circMID1 biogenesis. The knockdown of S100A9 significantly increased DHX9 protein expression levels in MDSC-Exo-treated PC3 and DU145 cells (Fig. 3D). DHX9 is a well-known RNA helicase that can interact with inverted Alu repeats (IARs) to decrease the expression of a subset of circRNAs [29]. Abundant IARs are found within the MID gene locus, some of which are located around exons 7 to 8 (Fig. 3E). The results confirmed that circMID1 abundance increased after silencing DHX9 (Fig. 3F, G), suggesting that DHX9 could be a potential regulator.

Additionally, to study the internalization of MDSC-derived exosome S100A9 by PCa cells, MDSC-Exo was labeled with carboxyfluorescin diacetate succinimidyl ester (CFSE) fluorescent dye. The uptake of MDSC-Exo S100A9 was observed using a confocal microscope. We found that CFSE-labeled exosomes were localized in the cytoplasm, the uptake of CFSE exosomes by PCa cells was evident at 48 h, suggesting that the number of CFSE exosomes absorbed by PC3 and DU145 cells was observed, but not in the control cells treated with free CFSE dye or incubated unlabeled exosomes (Fig. 3H). The S100A9 levels in PC3 cells after 48 h incubation with CFSE exosomes was then detected (Fig. 3I), indicating that S100A9 was highly expressed in MDSC-Exo-treated PC3 and DU145 cells.

### CircMID1 functioned as a miR-506-3p sponge

**Fig. 4:**
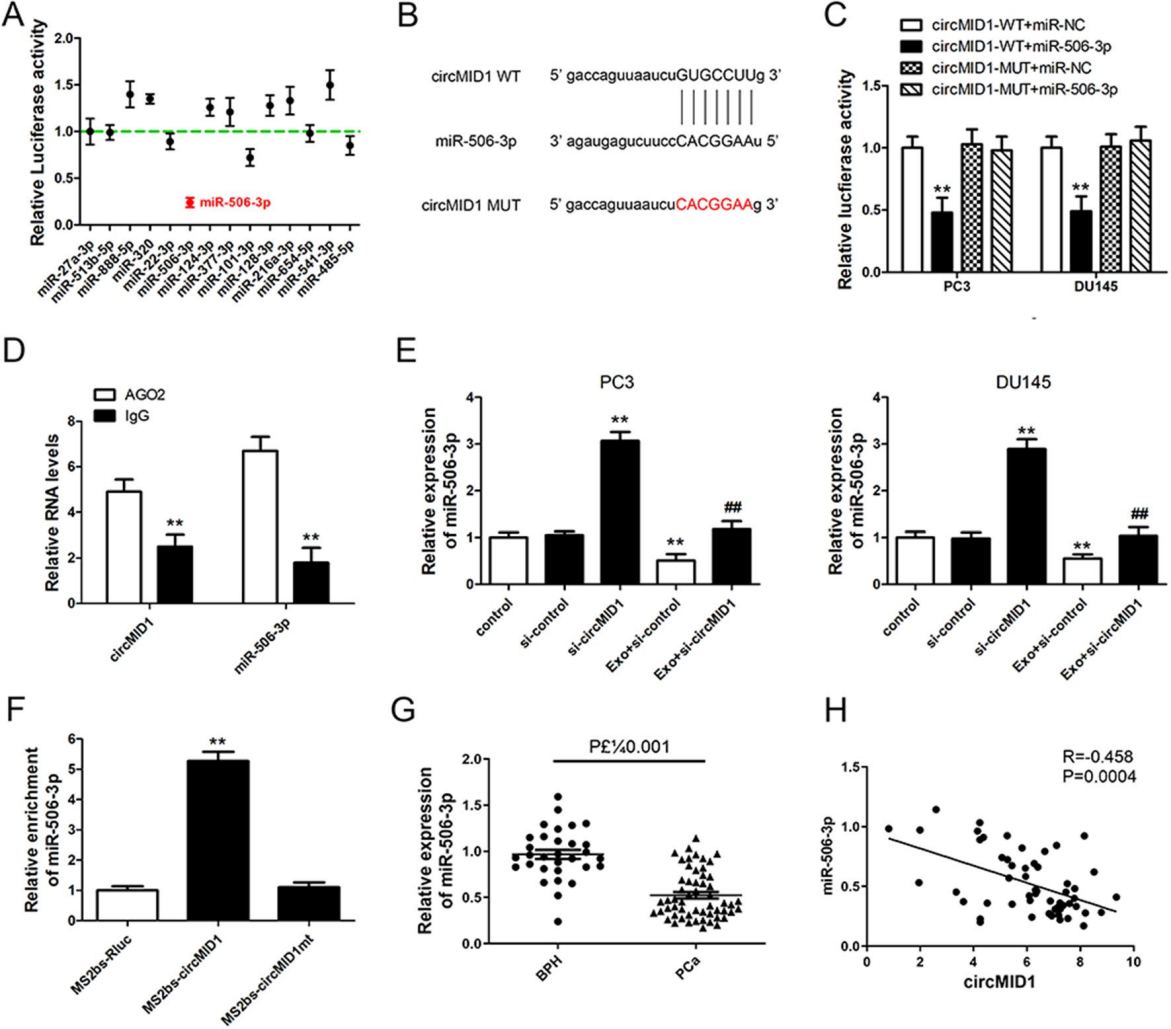
The circMID1 acts as a sponge for miR-506-3p. **A** HEK293 cells co-transfected with miRNAs mimics or control miRNAs (miR-NC) and luciferase reporter containing circMID1 (circMID1-WT) or mutant construct (circMID1-MUT). Dual luciferase reporter assays were performed. **B** The predicted binding sites of miR-506-3p and wild type (WT) or mutant (MUT, red) circMID1 are shown. **C** PC3 and DU145 cells co-transfected with miR-506-3p mimics or control miR-506-3p (miR-NC) and luciferase reporter containing circMID1 (circMID1-WT) or mutant construct (circMID1-MUT). Dual luciferase reporter assays were performed (^**^P < 0.01). ^*^ vs. circMID1-WT + miR-NC. **D** The Ago2 RIP showed that Ago2 significantly enriched circMID1 and miR-506-3p (^**^P < 0.01). **E** The expression level of miR-506-3p was determined by RT-PCR in PC3 and DU145 cells with si-control or si-circMID1 after MDSC-Exo treatments. ^**^, ^##^P < 0.01, ^*^ vs. control, # vs. Exo + si-control. **F** MS2bp-MS2bs based RIP assay in PC3 cells transfected with MS2bs-circMID1, MS2bs-circMID1mt, or MS2bs-Rluc (**G**) Relative expression of miR-506-3p in benign prostatic hyperplasia (BPH, n = 31) and PCa tissues (n = 56) determined by qRT-PCR. **H** Pearson’s correlation analysis showed a positive correlation between miR-506-3p and circMID1 (R = 0.458; P = 0.0004) in PCa tissues. All experiments were performed at least three times

To investigate the potential miRNAs associated with circMID1, the possible binding partners of circMID1 were predicted by bioinformatics analyses using two databases in the public domain: STARBASE version 3.0 and CIRCBASE. Bioinformatics data demonstrated that circMID1 interacted with several miRNAs including miR-27a-3p, miR-513b-5p, miR-888-5p, miR-320, miR-22-3p, miR-506-3p, miR-124-3p and so on. The luciferase reporter vector including the circMID1 sequence was constructed, which was transfected with various miRNA mimics into HEK293 cells. The results showed that miR-506-3p exclusively and significantly reduced fluorescein intensity (Fig. 4A). circMID1 had a binding site for miR-506-3p (Fig. 4B), suggesting that circMID1 had a stable interaction with miR-506-3p. Therefore, we conducted dual-luciferase reporter assays to determine whether miR-506-3p functioned as a circMID1 target in PCa cells. The luciferase activity was markedly reduced as cells were co-transfected with luciferase reporters, circMID1-WT and miR-506-3p mimics, meanwhile, co-transfection with mutated luciferase reporter circMID1-MUT and miR-506-3p mimics did not significantly affect luciferase activity in PC3 and DU145 cells (Fig. 4C). Previous results suggested that Argonaute 2 (Ago2) protein binds with miRNAs and circRNAs to form an RNA-induced silencing complex. We therefore performed RNA immunoprecipitation (RIP) assays to pull down RNA transcripts bound to Ago2 in PC3 cells. We found that miR-506-5p and circMID1 were pulled down efficiently by anti-Ago2, but not by the nonspecific anti-IgG antibody (Fig. 4D). Subsequently, we verified the circMID1 effect on PCa cell miR-506-3p expression. Knockdown results showed that si-circMID1 upregulated miR-506-3p expression in PC3 and DU145 cells without or with MDCS-Exo treatment (Fig. 4E). To further validate the direct binding between circMID1 and miR-506-3p, we performed MS2bp-MS2bs based RIP assay in PC3 cells. As shown in Fig. 4F, miR-506-3p was significantly enriched in RNAs retrieved from MS2bs-circMID1 compared with that from MS2bs-circMID1mt or control MS2bs-Rluc, indicating the specific interaction between circMID1 and miR-506-3p. Moreover, the miR-506-3p expression levels in the PCa samples were significantly lower than that of BPH tissues (Fig. 4G), and miR-506-5p was negatively associated with circMID1 in PCa tissues (Fig. 4H). The results agreed with the observed circMID1 overexpression in PCa and demonstrated that circMID1 was a miR-506-3p sponge in PCa.

### CircMID1 and MID1 functioned as ceRNAs in PCa through miR-506-3p regulation

**Fig. 5:**
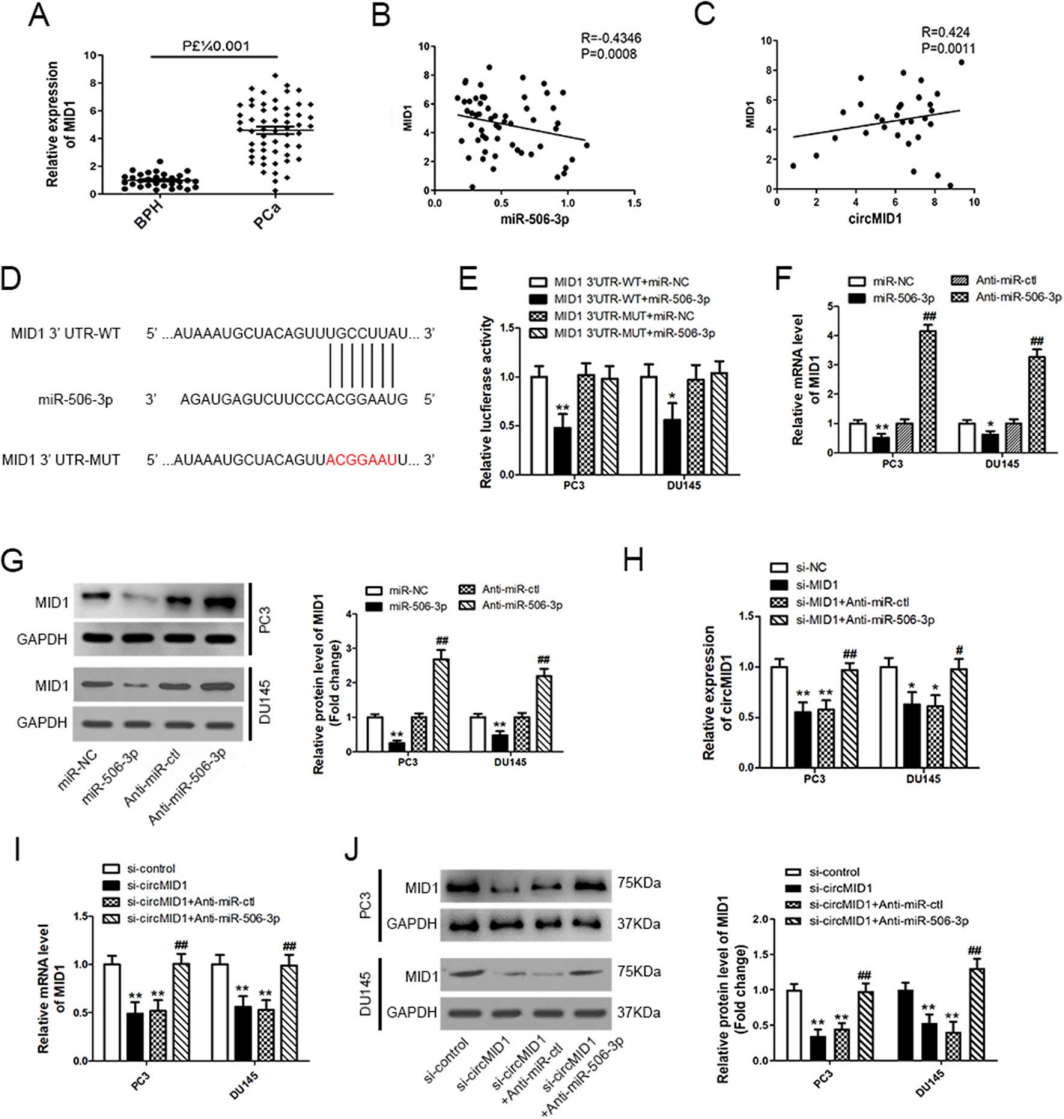
CircMID1 and MID1 act as ceRNAs in PCa through regulation of miR-506-3p. **A** The expression level of MID1 in benign prostatic hyperplasia (BPH, n = 31) and PCa tissues (n = 56) determined by qRT-PCR. (B-C) Pearson’s correlation analysis was used to analyze the relationships between MID1 mRNA and miR-506-3p (R = -0.4346, P = 0.0008, **B**), and MID1 mRNA and circMID1 (R = 0.424, P = 0.0011, **C**). **D** Potential binding site of miR-506-3p at the 3′-UTR of human MID1 mRNA is shown. Red color indicates the sequence of the mutated miR-506-3p-binding site. **E** PC3 and DU145 cells were cotransfected with miR-506-3p mimic or control mimic and luciferase reporter vector containing wild-type (WT) or mutated miR-506-3p-binding site (MUT) at MID1 3′-UTR. Luciferase reporter assays were performed. ^*^P < 0.05; ^**^P < 0.01, ^*^ vs. MID1 3′-UTR-WT + miR-NC. **F**, **G** PC3 and DU145 cells were transfected with miR-506-3p mimic or anti-miR-506-3p and their corresponding controls (miR-NC or anti-miR-ctl), and the mRNA (**F**) and the protein (**G**) expression levels of MID1 were analyzed by qRT-PCR and western blotting, respectively. ^*,#^P < 0.05; ^**,##^P < 0.01, ^*^ vs. miR-NC, ^#^ vs. Anti-miR-ctl. **H** PC3 and DU145 cells were transfected with si-NC, si-MID1, si-MID1 and anti-miR-ctl or anti-miR-506-3p, and the expression of circMID1 was determined by qRT-PCR. ^*,#^P < 0.05; ^**,##^P < 0.01, ^*^ vs. si-NC, ^#^ vs. si-MID1. **I**, **J** PC3 and DU145 cells were transfected with si-control, si-circMID1, or si-circMID1 and Anti-miR-ctl or Anti-miR-506-3p, and the mRNA (**I**) and protein (**J**) expression of MID1 were determined by qRT-PCR and western blotting, respectively. All experiments were conducted at least three times (^**^, ^##^P < 0.01). ^*^ vs. si-control, ^#^ vs. si-circMID1

To detect if circMID1 acted as a ceRNA to liberate the MID1 expression and sequester miR-506-3p, we then characterized MID1 expression in PCa tissues. The data verified that MID1 was upregulated in PCa tissues (Fig. 5A). Pearson’s correlation illustrated that MID1 was negatively correlated with miR-506-3p and positively correlated with circMID1 (Fig. 5B, C). In addition, we employed TargetScan to indicate the miR-506-3p putative target genes, which showed that MID1 was predicted (Fig. 5D). To verify this finding, we performed luciferase reporter assays, which showed that luciferase activity decreased significantly when being co-transfected with miR-506-3p mimics and a luciferase reporter containing WT MID1 3′-UTR, but not with a mutant luciferase reporter in PC3 and DU145 cells (Fig. 5E). Western blotting and qRT-PCR verified that MID1 protein and mRNA expression levels were decreased by miR-506-3p mimics and increased with miR-506-3p depletion by its antagomir in PC3 and DU145 cells (Fig. 5F, G). Together, these results suggested that MID1 was a direct miR-506-3p target, which might also sequester miR-506-3p.

We then characterized circMID1 expression after MID1 knockdown and found circMID1 repression (Fig. 5H). To validate if MID1 acted as a ceRNA, we co-transfected si-MID1 and miR-506-3p inhibitors and discovered that circMID1 repression was reversed in PC3 and DU145 cells (Fig. 5H). We then detected MID1 mRNA and protein expressions after circMID1 knockdown and observed reduced expression of both. The expression was reversed after co-transfection with miR-506-3p inhibitors in PC3 and DU145 cells (Fig. 5I, J). These data suggested that MID1 and circMID1 functioned as ceRNAs by harboring miR-506-3p.

### MDSC-Exo promoted prostate cancer cell proliferation, invasion, and migration by modulating S100A9/circMID1/miR-506-3p/MID1 signaling

**Fig. 6:**
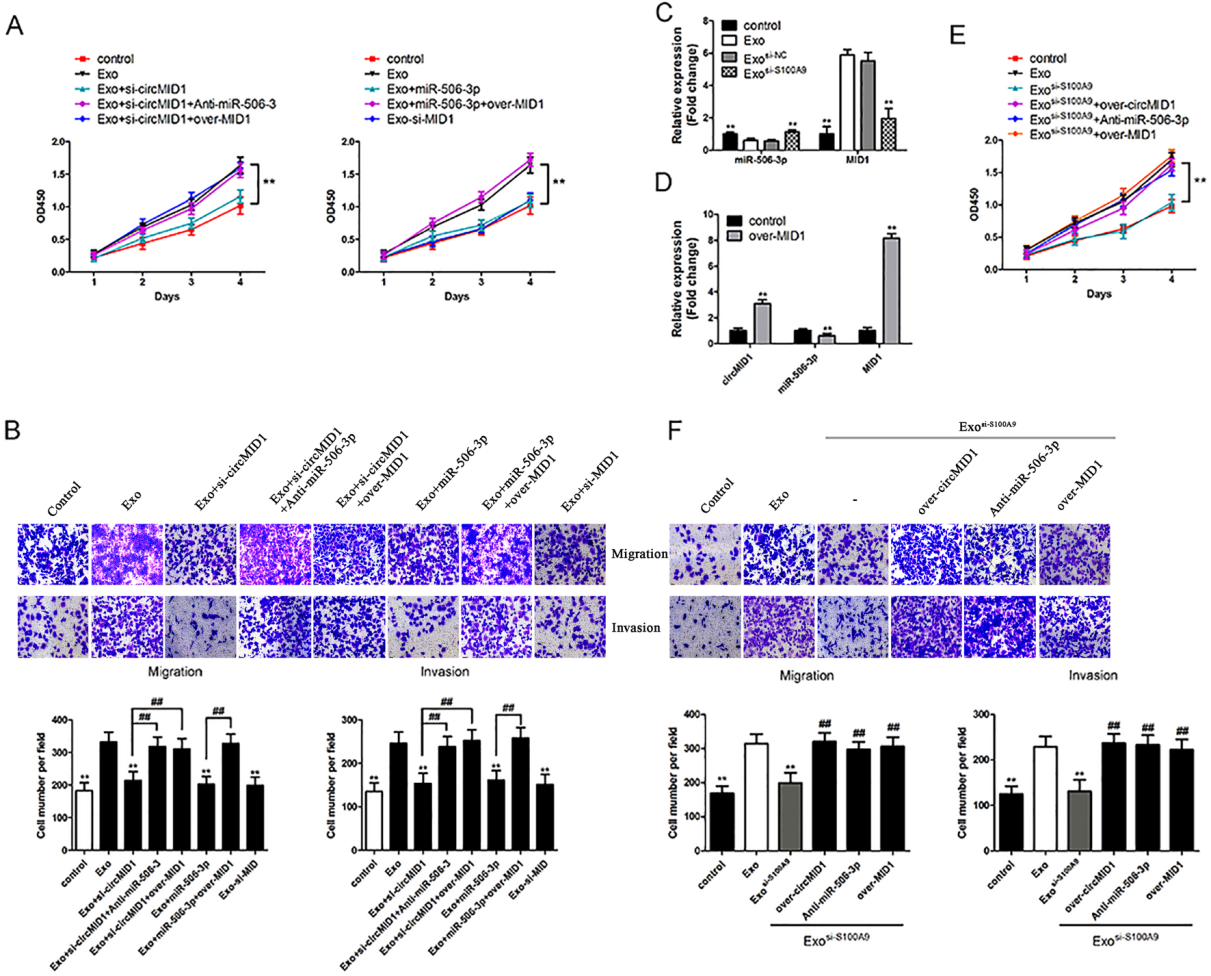
MDSC-Exo promoted PC3 cell proliferation, invasion, and migration by modulating S100A9/circMID1/miR-506-3p/MID1 signaling. **A**, **B** PC3 cells were transfected with si-circMID1, miR-506-3p, si-MID1, si-circMID1 and anti-miR-506-3p, or over-MID1, miR-506-3p and over-MID1, and then treated with MDSC-Exo. Cell proliferation (**A**) was measured by the CCK-8 assay in the PC3 cells with different treatments. Cell migration and invasion (**B**) was evaluated by Transwell assays of the PC3 cells with the indicated treatments. Scale bar, 100 μM. **C** Relative expressions of miR-506-3p and MID1 in PC3 cells treated with MDSC-Exo or MDSC-Exo^si−S100A9^. ^**^P < 0.01, ^*^ vs. Exo. **D** Relative expressions of circMID1, miR-506-3p and MID1 in PC3 cells transfected with over-MID1. ^**^P < 0.01, ^*^ vs. control. **E**, **F** PC3 cells were transfected with over-circMID1, anti-miR-506-3p and over-MID1, and then treated with MDSC-Exo^si−S100A9^. Cell proliferation (**E**) was measured by the CCK-8 assay, and cell migration and invasion (**F**) were evaluated by Transwell assays of these PC3 cells with the indicated treatments. ^**,##^P < 0.01, ^*^ vs. Exo, ^#^ vs. Exo^si−S100A9^. All experiments were conducted at least three times

To determine the effects of the circMID1/miR-506-3p/MID1 pathway on MDSC-Exo-regulated CRPC propagation, the circRNA-miRNA-mRNA axis was blocked or overexpressed through knockdown in PC3 cells treated with MDSC-Exo, and then analyzed by conducting functional assays. Data demonstrated that circMID1 knockdown or miR-506-3p overexpression or MID1 knockdown inhibited the proliferation, invasion, and migration of MDSC-Exo-treated PC3 cells, while miR-506-3p inhibition or MID1 overexpression rescued the circMID1-deficient or miR-506-3p-overexpressed cell proliferation, invasion, and migration defects after treatment with MDSC-Exo (Fig. 6A, B). In addition, we found that knockdown of S100A9 significantly increased the miR-506-3p levels and reduced MID1 expression levels in MDSC-Exo-treated PC3 cells (Fig. 6C). In MID1-overexpressed PC3 cells, the circMID1 expression levels were increased and the miR-506-3p levels reduced (Fig. 6D). The knockdown of S100A9 inhibited MDSC-Exo-treated PC3 cell proliferation, migration, and invasion, while circMID1 overexpression or miR-506-3p inhibition or MID1 overexpression rescued the inhibition effect of S100A9 knockdown (Fig. 6E, F). Moreover, we also found that in MDSC-Exo-treated DU145 cells the circMID1 and MID1 expression levels were increased and the miR-506-3p levels were reduced, while knockdown of S100A9 significantly increased the miR-506-3p levels and reduced circMID1 and MID1 expression levels (Additional file 2: Fig. S2A). The circMID1 knockdown or miR-506-3p overexpression or MID1 knockdown inhibited the proliferation, invasion, and migration of MDSC-Exo-treated DU145 cells (Additional file 2: Fig. S2B and S2D). The knockdown of S100A9 inhibited MDSC-Exo-treated DU145 cell proliferation, migration, and invasion, while circMID1 overexpression or miR-506-3p inhibition or MID1 overexpression rescued the inhibition effect of S100A9 knockdown (Additional file 2: Fig. S2C and S2E). Thus, the results showed that MDSC-Exo promoted prostate cancer cell migration, proliferation, and invasion through S100A9/circMID1/miR-506-3p/MID1 signaling.

### CircMID1 deficiency inhibited MDSC-Exo-induced prostate cancer growth in vivo

**Fig. 7:**
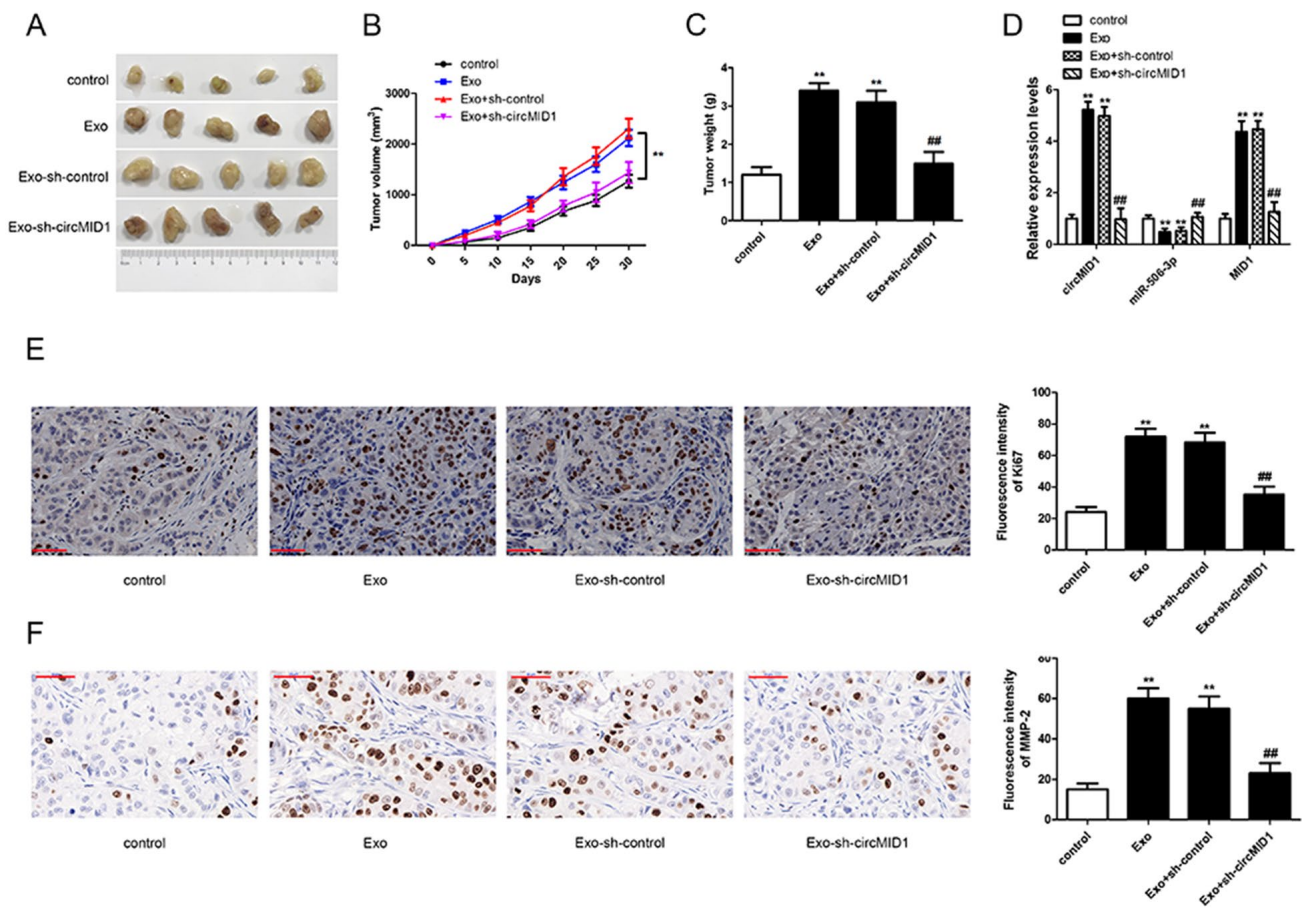
CircMID1 deficiency inhibited MDSC-Exo-induced prostate cancer growth in vivo*.* **A** Representative images of xenograft tumors in nude mice (n = 5/group). **B** The tumor volume was monitored every 5 days for 30 days. ^**^P < 0.01. **C** The tumor weight was determined at the end point of the xenograft experiment. ^**,##^P < 0.01, ^*^ vs. control, ^#^ vs. Exo. **D** The relative expressions of circMID1, miR-506-3p, and MID1 were analyzed by qRT-PCR in tumor tissues. **E** The expression of proliferation marker Ki67 in each group, detected by immunohistochemistry immunofluorescence (scale bar, 50 μM). **F** The expression of invasion-related factor MMP-2 in each group, detected by immunohistochemistry (scale bar, 50 μM). ^**,##^ P < 0.01, ^*^ vs. control, ^#^ vs. Exo. The measurement data were expressed as the mean ± standard deviation (SD). All experiments were repeated at least three times

Based on the previous results, the role of MDSC-Exo and circMID1 in PC3 tumor growth in vivo was evaluated by xenograft nude mice tumor formation assays. Nude mice were injected with control or MDSC-Exo-treated PC3 cells, or MDSC-Exo-PC3 cells transfected with si-control or si-circMID1, and tumor growth was measured. We found that MDSC-Exo promoted tumor growth, whereas knockdown of circMID1 caused a significant decrease in tumor growth (Fig. 7A–C). MDSC-Exo-upregulated circMID1 and MID1 expressions were inhibited by si-circMID1 in tumor tissues, while downregulated miR-506-3p expression was increased by si-circMID1 (Fig. 7D). Furthermore, immunohistochemical staining was carried out to determine the expression of the proliferation marker Ki67 and the invasion-associated factor MMP-2. The expression of Ki67 and MMP-2 in the Exo group was significantly increased compared to that in the control group; in comparison with the Exo group, the expression of Ki67 and MMP-2 in the Exo-sh-circMID1 group was significantly decreased (Fig. 7E, F). Together, these results indicated that silencing circMID1 inhibited the promotional effects of MDSC-Exo on prostate cancer growth via regulating signaling of miR-506-3p/MID1.

## Discussion

Evidence suggests that crosstalk between cancer cells and MDSCs induces cancer progression [31, 32]. MDSC-derived exosomes are found to function as intercellular messengers. Studies have analyzed the Exo activities derived from MDSCs under tumor environments [27, 33], but the underlying mechanisms remain poorly characterized. Our results showed, in agreement with previous findings of unbiased mass spectrometry analyses [27], that S100A9 was abundant in MDSC-Exo. Furthermore, the knockdown of S100A9 inhibited MDSC-Exo-treated PC3 cell migration, proliferation, and invasion. It has been reported that MDSC-derived Exos polarized macrophages to a phenotype that promoted tumor progression, and possessed S100A8/A9 chemotactic activity [27, 33], which suggested that MDSC shed exosomes, which functioned as communicators in the tumor microenvironment. Following ADT, most PCa develops into CRPC, which is a major clinical problem because the recurrent disease does not respond well to alternative therapies. Therefore, identification of the molecular mechanisms of androgen-independent signalling pathways using current genomic approaches would help to improve therapies for this disease.

So, the androgen independent PCa cell lines PC3 and DU145 were choose in this study. However, this can be a limitation. The existing research for understanding of this mechanism of PCa progression is not comprehensive due to lack of available data from one androgen dependent PCa cell line. This research analyzed the circRNA expression profiles in MDSC-Exo-PC3 cells compared with control cells by microarray analyses, and focused on the role and underlying mechanism of the increased circMID1 expression in MDSC-Exo-regulated CRPC progression. Collectively, our results suggested that exosome-mediated transfer of S100A9 from MDSCs to PCa cells promoted proliferation, migration, and invasion of PCa cells via upregulation of circMID1, ultimately accelerating the progression of CRPC.

CircRNAs, as a novel class of noncoding RNAs, are known to participate in cancer progression [34, 35]. Studies have shown that circAMOTL1L is downregulated in human PCa, and this downregulation promotes PCa cell migration and invasion, which leads to the epithelial–mesenchymal transition and PCa progression [36]. Huang et al. found that circABCC4 was upregulated in PCa cell lines and tissues, and facilitated PCa malignant behavior by promoting FOXP4 expression through miR-1182 sponging [37]. While the biological function and expression of circRNAs in CRPC are poorly understood, current studies have identified that circMID1 was upregulated in PC3 cells treated with Exos from MDSCs. Moreover, higher expression of circMID1 was found in PCa compared with BPH tissues and in CRPC patients compared with HSPC patients. Subsequent studies showed that circMID1 knockdown decreased cell proliferation and inhibited PC3 cell invasion and migration with MDSC-Exo. We further verified tumor growth in a mouse xenograft model. These findings supported the concept that increased circMID1 expression caused by MDSC-Exo might exert functional roles in the regulation of CRPC progression. Ding Y and coworkers have recently documented that the circMID1 is upregulated in PCa cells and that its knockdown inhibits PCa cell proliferation, migration, invasion in vitro, as well as PCa tumorigenesis in vivo [38], which is consistent with our findings. However, our study also has a limitation. Because we performed bioinformatics analysis to screen out circMID1 only using the two samples and there is a big variability between the two control samples, other important circRNAs may be ignored.

This study showed that circMID1 expression was decreased while DHX9 expression was increased in PC3 cells treated with S100A9-silenced MDSC-Exo. DHX9, an abundant nuclear RNA helicase, binds specifically to IARs, and loss of DHX9 increases the formation of a subclass of circRNAs [29, 39]. Alu elements are well-known retrotransposons, members of the short interspersed nuclear element (SINE) family of repetitive elements, and IARs around exons promote circRNA formation [40, 41]. We found that multiple IARs were located within the *MID1* gene, some of which were around exons 7 to 8, where circMID1 was formed. CircMID1 expression was further elevated by silencing DHX9, suggesting that DHX9 affected the circularization of the *MID1* gene in an Alu element-dependent manner.

The circRNA-miRNA-mRNA axis is an indispensable regulatory model and circRNAs could function as ceRNAs by regulating miRNAs to play a critical role in transcriptional control [22, 42]. Based on bioinformatics analyses, it was assumed that the circMID1/miR-506-3p/MID1 axis functioned essentially in MDSC-Exo-regulated CRPC progression. Bioinformatics analyses and luciferase reporter assays indicated that circMID1 sponged miR-506-3p, and MID1 was the miR-506-3p target. Cytological functional experiments validated that circMID1 and miR-506-3p could reverse the effects on the cell phenotype. A miR-506-3p antagomir could rescue biological alterations induced by circMID1 silencing. Specifically, we showed mechanistically that circMID1 was involved in MDSC-Exo-regulated CRPC progression by acting as a miR-506-3p sponge. However, circMID1 may also interact with other miRNAs according to bioinformatics analysis. Thus, whether other miRNAs have a role in circMID1-regulated tumorigenesis needs to be investigated in the future.

The upregulated circMID1 was accompanied by downregulated miR-506-3p and upregulated MID1 in PCa tissues. Studies have verified that MID1 is overexpressed in PCa in a stage-dependent manner, and AR is promoted by sustained MID1 upregulation in ADT, causing PCa progression into castration resistance [43]. In this study, MID1 was overexpressed in PCa, and miR-506-3p bound to MID1 and inhibited MID expression in PC3 cells. The circMID1 regulated MID1 expression by sponging miR-506-3p because miR-506-3p inhibition released the inhibitory effect of circMID1 deficiency upon MID1 expression. The circRNA enhanced PCa progression through MID1 because MID1 overexpression rescued the progression defect mediated by circMID1 deficiency in MDSC-Exo-PC3 cells. Furthermore, circMID1 overexpression or miR-506-3p inhibition or MID1 overexpression rescued the inhibitory effect of S100A9-knockdown MDSC-Exo on PC3 and DU145 cells. Thus, the study validated that a S100A9/circMID1/miR-506-3p/MID1 axis existed in MDSC-Exo-regulated CRPC progression.

## Conclusion

**Fig. 8:**
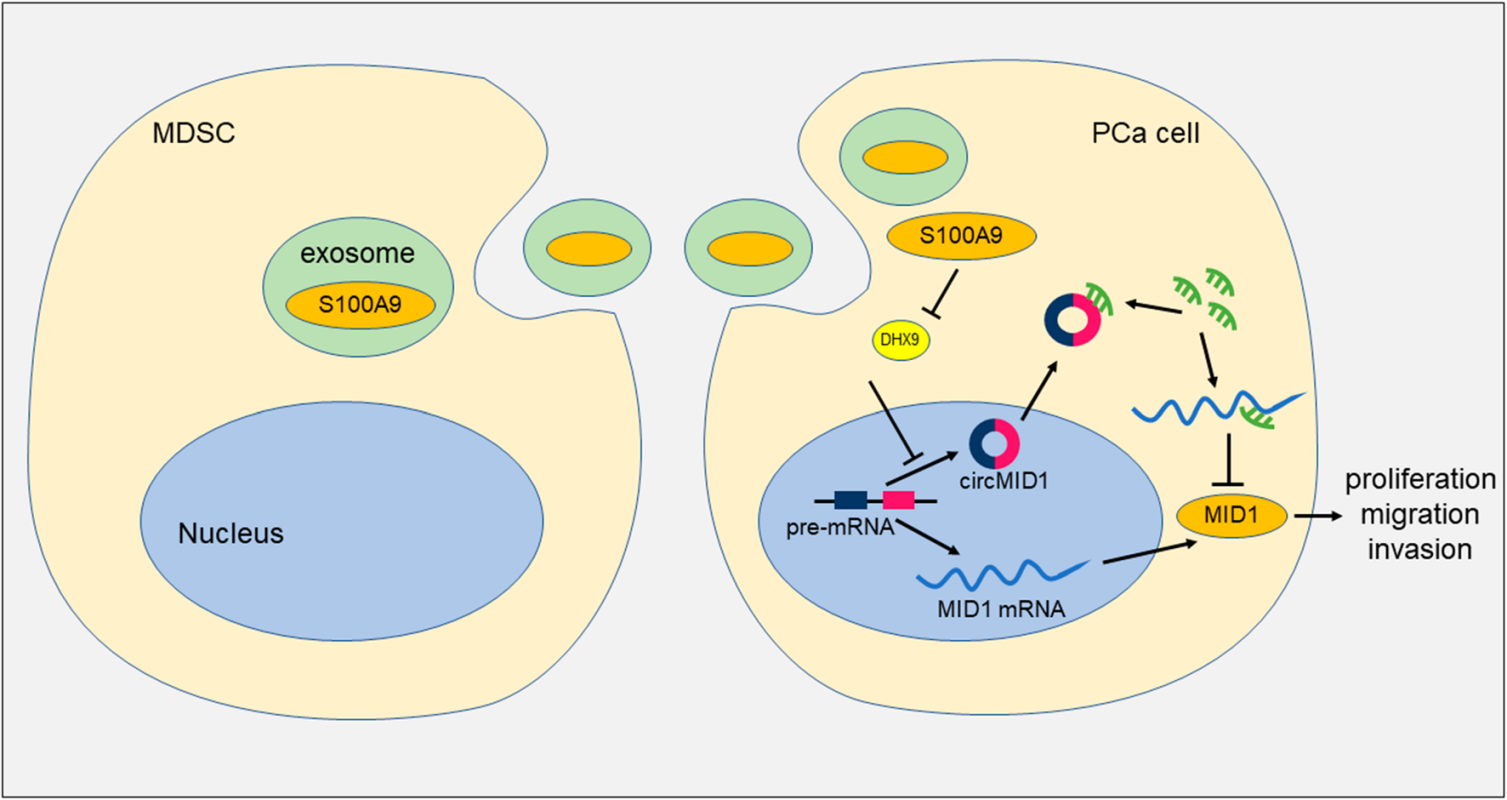
Mechanism of MDSC-Exo in prostate cancer progression by regulating circMID1. MDSC-derived exosomes (MDSC-Exo) accelerated PCa cell proliferation, migration, and invasion, while circMID1 (hsa_circ_0007718) expression was elevated in PCa cells treated with MDSC-Exo. Exosomes secreted from MDSCs could transfer S100A9 to prostate cancer cells, upregulating the expression circMID1 by inhibiting DHX9. MDSC-derived exosomal S100A9 increased circMID1 expression to sponge miR-506-3p, leading to increased MID1 expression and accelerated tumor progression

In conclusion, our data suggested that circMID1 was upregulated in MDSC-Exo-regulated CRPC progression, and that circMID1 knockdown inhibited growth and prostate cancer cell invasion and migration, with MDSC-Exo. The identification of the novel alteration in circRNAs expression is the first key step toward the better understanding of the role of circRNAs in CRPC. Then, deciphering the exact molecular mechanisms of circRNA function will be critical for understanding CRPC progression and developing new potential therapeutic targets. Exosomal S100A9 from MDSC promoted circMID1 expression in PC3 cells, and the circMID1 acted as a ceRNA to regulate MID1 expression through miR-506-3p (Fig. 8). We discovered an essential S100A9/circMID1-miR-506-3p-MID1 axis in MDSC-Exo-regulated CRPC progression and found a critical function of circRNAs in disease progression.

## Supplementary Information

**Additional file 1: Figure S1.** Identification of MDSC-exosomes. (A) Dot plots representing the gating and sorting strategy of PMN-MDSCs (green box) of PBMCs. (B) Representative transmission electron micrograph of MDSC-Exo (scale bar = 200 nm). (C) The CD9, CD63, Hsp70 and calnexin expression levels of MDSC-Exo were detected by western blotting. (D) Analysis of size and concentration of exosomes by nanoparticle tracking analyzer.

**Additional file 2: Figure S2.** MDSC-Exo promoted DU145 cell proliferation, invasion, and migration by modulating S100A9/circMID1/miR-506-3p/MID1 signaling. (A) Relative expressions of circMID1, miR-506-3p and MID1 in DU145 cells treated with MDSC-Exo or MDSC-Exosi-S100A9. ** P < 0.01, * vs. Exo. (B) DU145 cells were transfected with si-circMID1, miR-506-3p and si-MID1, and then treated with MDSC-Exo. Cell proliferation was measured by the CCK-8 assay in the DU145 cells with different treatments. (C) DU145 cells were transfected with over-circMID1, anti-miR-506-3p and over-MID1, and then treated with MDSC-Exo^si−S100A9^. Cell proliferation was measured by the CCK-8 assay. (D-E) Cell migration and invasion was evaluated by Transwell assays of these DU145 cells with the indicated treatments. Scale bar, 100 μM. **, ## P < 0.01, * vs. Exo, # vs. Exo^si−S100A9^. All experiments were conducted at least three times.

## Data Availability

The datasets used and/or analyzed during the current study are available from the corresponding author on reasonable request.

## Abbreviations

CRPC: Castration-resistant prostate cancer
PCa: Prostate cancer
MDSCs: Myeloid-derived suppressor cells
Exos: Exosomes
BPH: Benign prostatic hyperplasia
HSPC: Hormone sensitive prostate cancer
TME: Tumor microenvironments
AR: Androgen receptor
circRNAs: Circular RNAs
GAPDH: Glyceraldehyde 3-phosphate dehydrogenase
Ago2: Argonaute 2
RIP: RNA immunoprecipitation
