# Supplementary figures and images for: Exosomes derived from myeloid-derived suppressor cells facilitate castration-resistant prostate cancer progression via S100A9/circMID1/miR-506-3p/MID1
Source: J Transl Med. 2022 Aug 2;20:346. doi: 10.1186/s12967-022-03494-5 (PMC9344715; doi:10.1186/s12967-022-03494-5)

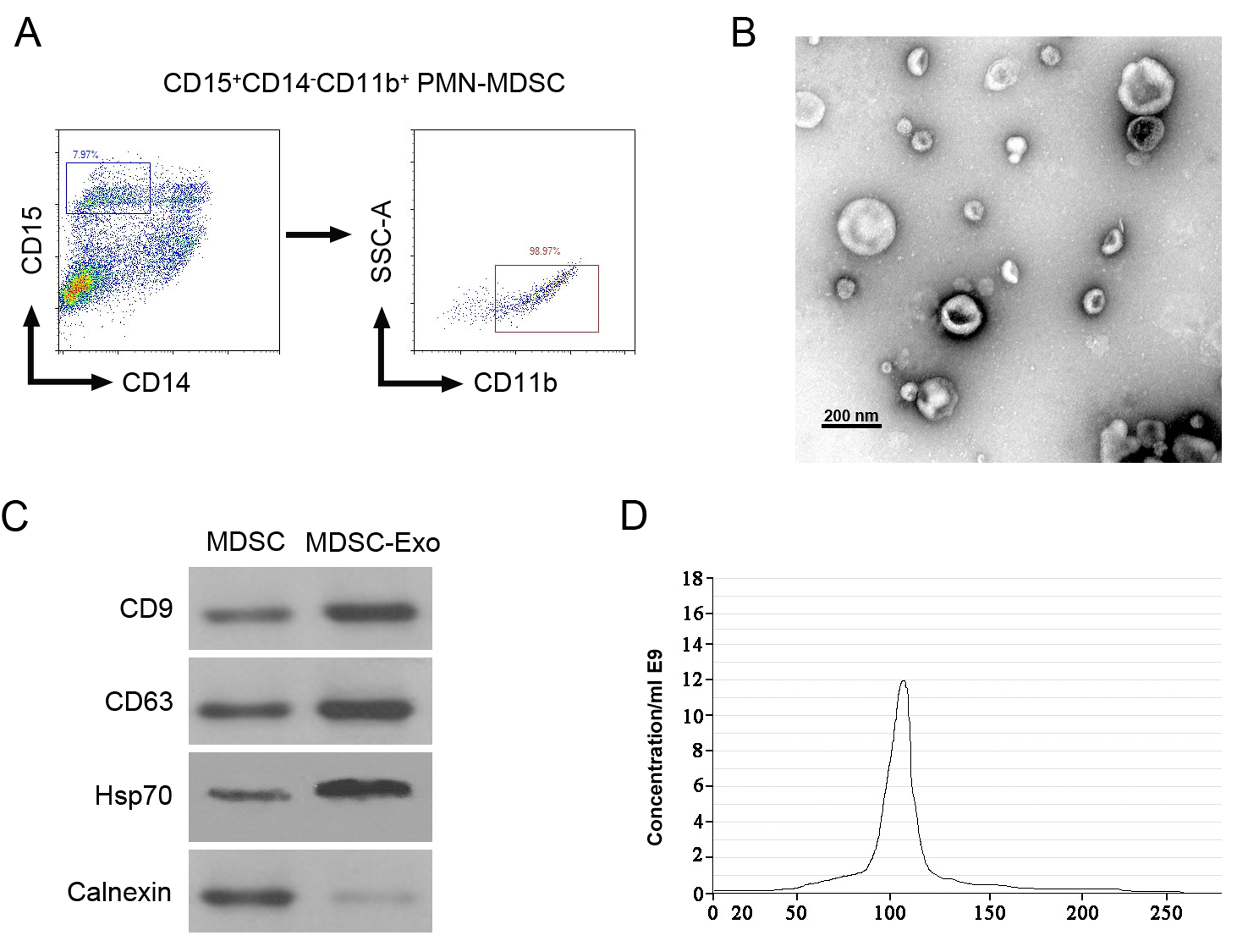

Supplement: Supplementary file 1 — Additional file 1: Figure S1. Identification of MDSC-exosomes. (A) Dot plots representing the gating and sorting strategy of PMN-MDSCs (green box) of PBMCs. (B) Representative transmission electron micrograph of MDSC-Exo (scale bar = 200 nm). (C) The CD9, CD63, Hsp70 and calnexin expression levels of MDSC-Exo were detected by western blotting. (D) Analysis of size and concentration of exosomes by nanoparticle tracking analyzer. [file 12967_2022_3494_MOESM1_ESM.tif]

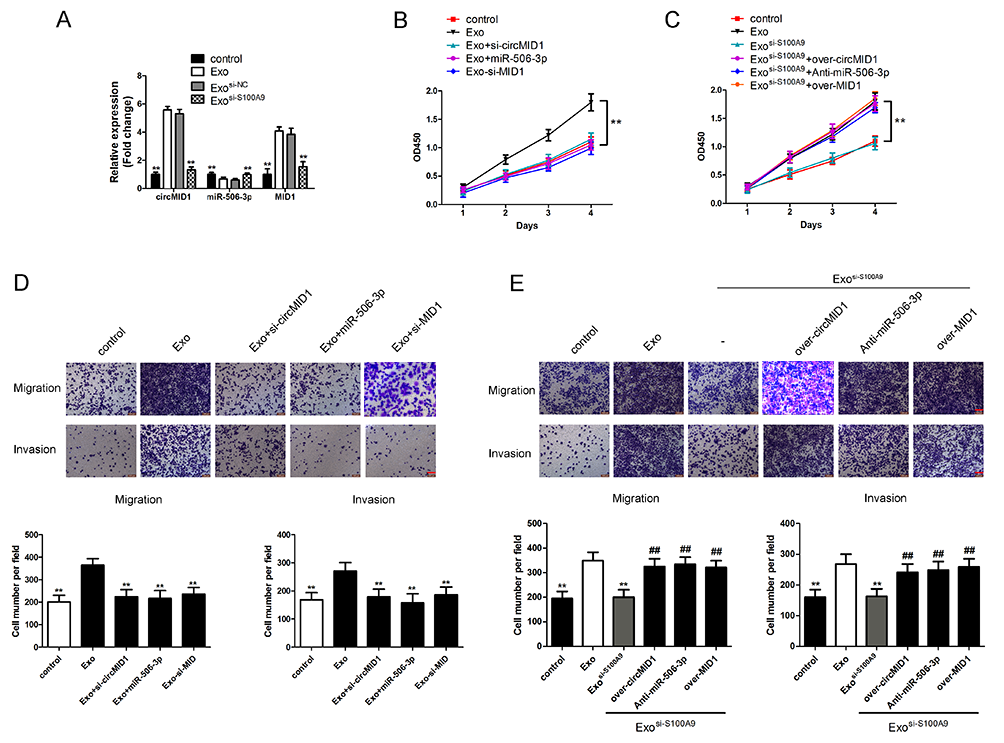

Supplement: Supplementary file 2 — Additional file 2: Figure S2. MDSC-Exo promoted DU145 cell proliferation, invasion, and migration by modulating S100A9/circMID1/miR-506-3p/MID1 signaling. (A) Relative expressions of circMID1, miR-506-3p and MID1 in DU145 cells treated with MDSC-Exo or MDSC-Exosi-S100A9. ** P < 0.01, * vs. Exo. (B) DU145 cells were transfected with si-circMID1, miR-506-3p and si-MID1, and then treated with MDSC-Exo. Cell proliferation was measured by the CCK-8 assay in the DU145 cells with different treatments. (C) DU145 cells were transfected with over-circMID1, anti-miR-506-3p and over-MID1, and then treated with MDSC-Exosi−S100A9. Cell proliferation was measured by the CCK-8 assay. (D-E) Cell migration and invasion was evaluated by Transwell assays of these DU145 cells with the indicated treatments. Scale bar, 100 μM. **, ## P < 0.01, * vs. Exo, # vs. Exosi−S100A9. All experiments were conducted at least three times. [file 12967_2022_3494_MOESM2_ESM.tif]
